# Red Light-Emitting Water-Soluble Luminescent Iridium-Containing Polynorbornenes: Synthesis, Characterization and Oxygen Sensing Properties in Biological Tissues In Vivo

**DOI:** 10.3390/molecules26216349

**Published:** 2021-10-20

**Authors:** Leonid N. Bochkarev, Yulia P. Parshina, Yana V. Gracheva, Tatyana A. Kovylina, Svetlana A. Lermontova, Larisa G. Klapshina, Aleksey N. Konev, Mikhail A. Lopatin, Maria M. Lukina, Anastasia D. Komarova, Vladislav I. Shcheslavskiy, Marina V. Shirmanova

**Affiliations:** 1 Razuvaev Institute of Organometallic Chemistry, Russian Academy of Sciences, Tropinina, 49, 603950 Nizhny Novgorod, Russia; jully@iomc.ras.ru (Y.P.P.); yanashlyapugina@yandex.ru (Y.V.G.); gluhova@iomc.ras.ru (T.A.K.); lermontovasa@rambler.ru (S.A.L.); klarisa@iomc.ras.ru (L.G.K.); alex-kon@mail.ru (A.N.K.); lopatin@iomc.ras.ru (M.A.L.); 2 Institute of Experimental Oncology and Biomedical Technologies, Privolzhsky Research Medical University, Minin and Pozharsky Sq. 10/1, 603005 Nizhny Novgorod, Russia; kuznetsova.m.m@yandex.ru (M.M.L.); leeemonk76g@gmail.com (A.D.K.); vis@becker-hickl.de (V.I.S.); shirmanovam@mail.ru (M.V.S.); 3 Becker&Hickl GmbH, Nunsdorfer Ring 7-9, 12277 Berlin, Germany

**Keywords:** iridium-containing polynorbornenes, bioimaging, phosphorescence lifetime imaging, oxygen sensing, tumor, in vivo

## Abstract

New water-soluble polynorbornenes **P1**–**P4** containing oligoether, amino acid groups and luminophoric complexes of iridium(III) were synthesized by ring-opening metathesis polymerization. The polymeric products in organic solvents and in water demonstrate intense photoluminescence in the red spectral region. The polymers **P1** and **P3** with 1-phenylisoquinoline cyclometalating ligands in iridium fragments reveal 4–6 fold higher emission quantum yields in solutions than those of **P2** and **P4** that contain iridium complexes with 1-(thien-2-yl)isoquinoline cyclometalating ligands. The emission parameters of **P1**–**P4** in degassed solutions essentially differ from those in the aerated solutions showing oxygen-dependent quenching of phosphorescence. Biological testing of **P1** and **P3** demonstrates that the polymers do not penetrate into live cultured cancer cells and normal skin fibroblasts and do not possess cytotoxicity within the concentrations and time ranges reasonable for biological studies. In vivo, the polymers display longer phosphorescence lifetimes in mouse tumors than in muscle, as measured using phosphorescence lifetime imaging (PLIM), which correlates with tumor hypoxia. Therefore, preliminary evaluation of the synthesized polymers shows their suitability for noninvasive in vivo assessments of oxygen levels in biological tissues.

## 1. Introduction

Amphiphilic polymers of different structure and composition are known as one of the most efficient systems for the delivery of therapeutic and diagnostic agents [1,2,3,4]. The use of luminescent metal-containing complexes in polymers allows the development of polymeric bioimaging agents with desirable spectral characteristics. Several representatives of water-soluble metal-containing polymers have been synthesized and successfully used as bioimaging and sensing agents [5,6,7,8,9]. These polymers have been prepared via Suzuki coupling polymerization [5,6], free-radical polymerization [7,8] and ring-opening metathesis polymerization (ROMP) [9]. Among the abovementioned polymerization methods, ROMP is the most suitable polymerization route because ROMP reactions proceed rapidly in the presence of air-stable and functional groups of tolerant ruthenium Grubbs catalysts in mild conditions and allow the acquisition of different functional polymeric materials with desired structure and composition [10,11,12,13]. The employment of ROMP for the preparation of polymeric materials for different biomedical applications is presented in recent articles [14,15,16]. Hango et al. developed oxanorbornene-based block copolymers capable of effective intracellularly protein delivery [14]. In the paper by Kockelmann et al., 2020, it is reported that poly(norbornene)-based nanogels can be utilized as drug delivery agents in anticancer immunotherapy [15]. A dendron-functionalized norbornene monomer was used for the preparation of dendronized polymers applicable as siRNA delivery systems [16].

Cyclometalated iridium(III) complexes are attractive luminophoric groups in amphiphilic polymers. Luminescent iridium complexes are currently among the most efficient photo- and electroluminophores and are widely applied as emitting materials in organic light-emitting diodes [17,18,19], bioimaging agents [20,21,22] and photocatalysts [23]. The luminescent efficiency and the emission color largely depend on the nature of ligands attached to iridium. The variation of ligands allows the modification of the spectral characteristics of iridium luminophores [17,18,19]. Iridium complexes emitting in the red and near-infrared (NIR) regions (called the ‘‘biological transparency window’’) are the most valuable for bioimaging and sensing applications. Red and NIR radiation penetrates deeply into biological tissues and can be easily discriminated from the background fluorescence of the biological environment. The general approach to achieve the desirable red and NIR emission consists of incorporating cyclometalating ligands with an extended conjugated aromatic system such as 1-phenylisoquinoline, 1-(benzo[b]thiophen-2-yl)isoquinoline, 6-(benzo[b]thiophen-2-yl)phenanthridine into iridium(III) complexes [8,24]. Iridium complexes with oxygen-dependent photophysical properties (e.g., emission intensity or lifetime) are of special interest for biomedical tasks related to oxygen assessment. For example, oxygen content is an important factor in the regulation of cancer metabolism and response to therapy [25,26]. A lack of oxygen promotes aggressive behavior of tumor cells and reduces the efficacy of oxygen-consuming therapies, such as photodynamic therapy and radiotherapy. Therefore, noninvasive sensing of tumor oxygenation would be advantageous to monitor treatment progress and predict outcome.

In the last decade, a number of iridium luminophores have been synthesized and their use as phosphorescent oxygen sensors in tumor cells and tissues has been demonstrated [22,27,28,29]. Neutral and ionic deep red emitting iridium(III) complexes have been obtained recently and their applicability as oxygen-sensing probes in live cells and tissues was investigated in detail [30,31]. Meanwhile, the search for new sensors that are more suitable for biological applications is still in demand to meet the requirements of high water solubility, low toxicity, high phosphorescence quantum yield and strong dependence of phosphorescence signal on oxygen concentration in the physiological range. Furthermore, oxygen measurements in tissues impose additional requirements: the probe should be either impermeable to biological membranes or preserve stable localization within the cell to avoid the influence of a complex and heterogeneous intracellular environment.

Herein, we report the preparation and photophysical and oxygen-sensing properties of new water-soluble luminescent iridium-containing polynorbornenes **P1**–**P4** emitting in the red spectral region. The polymers **P1**–**P4** were synthesized by the ROMP method because of its obvious advantages in comparison with other polymerization methods such as free-radical polymerization and Suzuki coupling polymerization. In our previous research [32], we demonstrated that 7-oxa-norbornene monomers with oligoether, amino acid groups and norbornene monomers with iridium complexes containing 2-phenylpyridine, 2-(2,4-difluorophenyl)pyridine, 2-(benzo[*b*]thiophen-2-yl)pyridine cyclometalating ligands could be used for the preparation of water-soluble polymers that exhibited photoluminescence in the green, blue-green and red spectral regions. The polymers **P1**–**P4** synthesized in the present study revealed far-red luminescence with higher quantum yields and enhanced oxygen sensitivity compared to the red-emitting polymers obtained previously. The improved photophysical characteristics of new iridium-containing polymers **P1** and **P3** allowed us to apply them for in vivo assessments of the oxygen level in cancerous and normal tissues.

## 2. Results and Discussion

### 2.1. Synthesis of Polymers ***P1***–***P4***

**Scheme 1:**
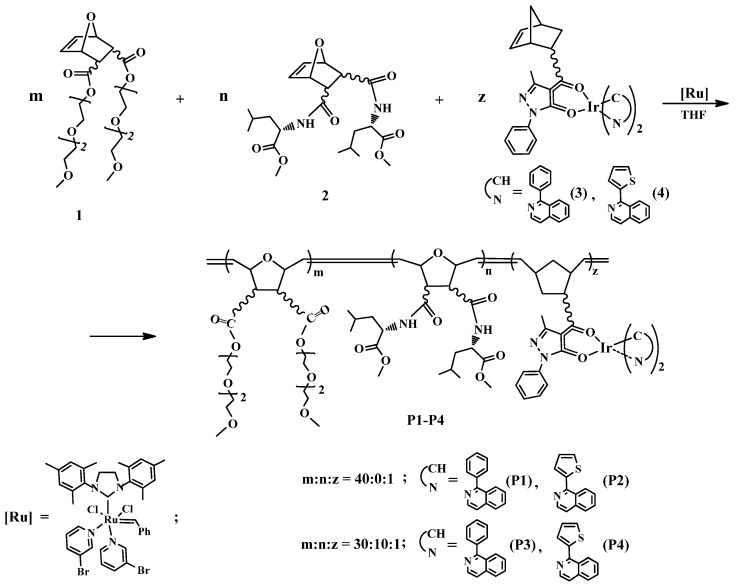
Synthesis of iridium-containing polymers **P1**–**P4**.

The ROMP method was applied for the preparation of the titled polymers **P1**–**P4**. The ROMP reactions proceed, as a rule, in a controllable fashion and allow the acquisition of various functionalized polymers with desirable molecular-weight characteristics [10,11,12,13]. The synthesis of iridium-containing polymers is presented in Scheme 1.

Organic monomer **1** was used for the preparation of water-soluble polymers **P1**–**P4** because it was known that ROMP polymers on the base of norbornene monomers with oligoether groups are soluble in water [33]. The monomer **2** was used for introduction into polymers **P3** and **P4** with additional units with amino acid fragments that might facilitate the address delivery of iridium-containing polymers to biological targets. In our previous research, we found that the iridium-containing monomers **3** and **4** entered into ROMP and formed polymeric materials revealing intense red luminescence [34,35]. Therefore, we used monomers **3** and **4** for the preparation of water-soluble polymers exhibiting luminescence in the red spectral region.

**Figure 1:**
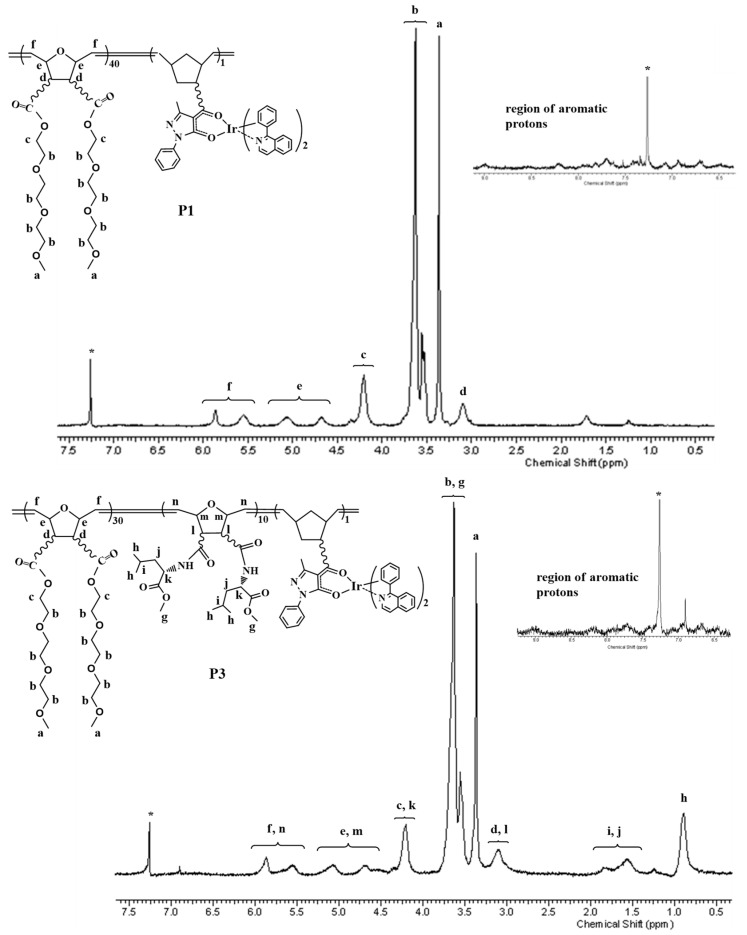
^1^H NMR spectra of polymers **P1**, **P3** in CDCl_3_. * Signal derived from the solvent.

Organic monomers **1** and **2** were copolymerized with iridium-containing monomers **3** and **4** in the presence of the third-generation Grubbs catalyst (monomers/catalyst = 100/1). ROMP reactions were monitored by thin layer chromatography and found to reach completion in 10 h at 40 °C. Isolated polymers **P1**–**P4** are red gummy substances soluble in THF, CH_2_Cl_2_, DMSO, EtOH and H_2_O and insoluble in hexane. After the exposure of the polymeric products in air for a month, their physico-chemical and photophysical characteristics remained unchanged. Elemental analysis, IR and ^1^H NMR spectroscopy data (experimental part, Figure 1 and Figure S1) confirmed the composition of polymers **P1**–**P4** shown in Scheme 1. The ratio of peak areas of aromatic, olefinic and aliphatic protons in the ^1^H NMR spectra is in accordance with the chemical structures of the synthesized polymers.

**Figure 2:**
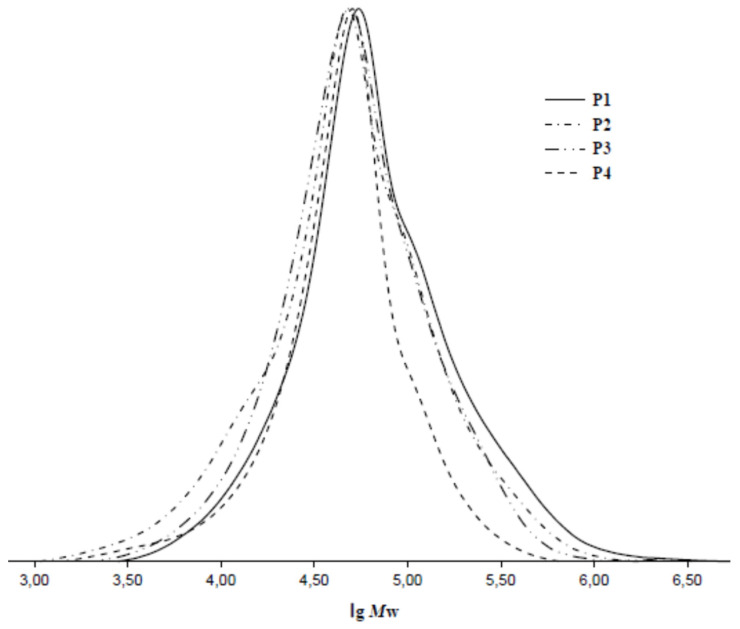
Normalized GPC curves of polymers **P1**–**P4**.

GPC analysis (Figure 2) revealed that average molecular weights and molecular-weight distributions of polymers **P1**–**P4** (*M*_w_ = 88,600–123,300, *M*_w_/*M*_n_ = 1.56–2.05) were comparable with appropriate characteristics of known ROMP polymers based on monomers **1** and **2** [36,37,38].

**Figure 3:**
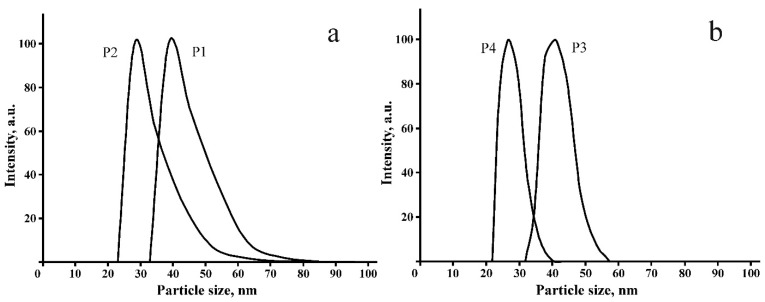
Particle size distribution of polymers **P1**, **P2** (**a**) and **P3**, **P4** (**b**) in aqueous solutions.

In aqueous solutions at a concentration of 0.1–0.2 g/L, polymeric products form nanoparticles with average sizes of 39 nm (**P1)**, 28 nm (**P2)**, 41 nm (**P3)** and 27 nm (**P4)**. The particle size distribution of **P1**–**P4** determined by the dynamic light scattering method is shown in Figure 3. It can be assumed that these particles are micelles, the shell of which consists of oligoether groups and amino acid fragments, and the core includes side chains with iridium complexes.

### 2.2. Photophysical Properties of Polymers ***P1***–***P4***

**Figure 4:**
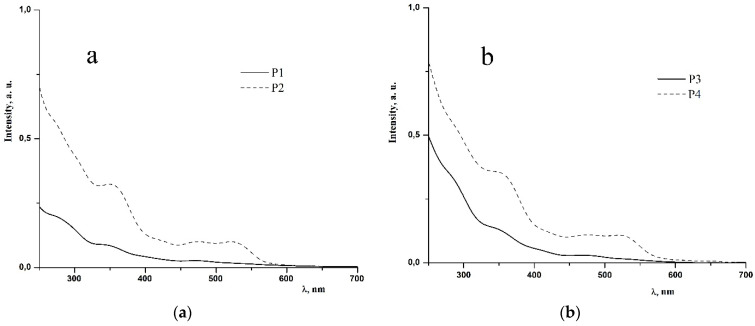
Absorption spectra of **P1**, **P2** (**a**) and **P3**, **P4** (**b**) polymers in CH_2_Cl_2_ solution.

Absorption spectra of **P1**–**P4** copolymers in methylene chloride and water are similar and contain intense bands in the region of 260–350 nm due to π→π* transitions in aromatic systems of ligands linked to iridium and low-intensity bands in the region of 360–550 nm corresponding to metal-to-ligand charge transfer transitions (MLCT) (Figure 4 and Figure S2 and Table 1 and Table S1) [34,35].

It should be noted that polymers **P2** and **P4** containing iridium complexes with 1-(thien-2-yl)isoquinoline ligands possess higher absorption ability than the polymers **P1** and **P3** that contain complexes with 1-phenylisoquinoline ligands (Table 1 and Table S1). Similar differences in the absorption ability were found earlier for the homoligand iridium complexes Ir(tiq)_3_ and Ir(piq)_3_ with 1-(thien-2-yl)isoquinoline and 1-phenylisoquinoline ligands accordingly [39].

**Figure 5:**
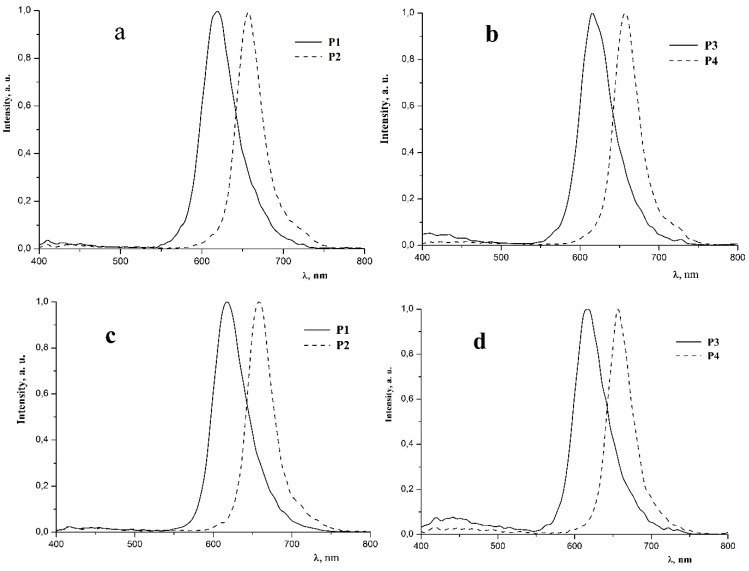
Normalized PL spectra of **P1**–**P4** polymers in (**a**,**b**) degassed CH_2_Cl_2_ solution and (**c**,**d**) degassed aqueous solution at room temperature, λ_ex_ = 360 nm.

In the photoluminescence (PL) spectra of **P1**–**P4** polymers in methylene chloride and water (Figure 5, Table 1 and Table S1), wide bands appear with maxima at 618 (**P1**, **P3**) and 657 nm (**P2**, **P4**) associated with ^3^MLCT and ligand-centered (^3^LC) transitions in cyclometalated iridium complexes linked to the polymer chain [34,35,39]. The chromaticity coordinates of PL spectra of **P1**–**P4** polymers in the CIE (Commission Internationale de l’Eclairage) diagram (Table 1) correspond to the red color. The PL characteristics of the synthesized polymers are mainly determined by the nature of cyclometalated iridium complexes incorporated into the macromolecules. The polymer **P1** based on iridium-containing monomer **3** and organic monomer **1** with oligoether groups and polymer **P3** containing additional units with amino acid fragments possess similar PL parameters. The same feature was found for polymers **P2** and **P4** based on iridium monomer **4**. These findings reveal that incorporation into polymer units with amino acid fragments does not noticeably change their PL properties.

The quantum yields of the PL of polymeric products in degassed CH_2_Cl_2_ solution (Table 1) are appreciably larger than the quantum yields in the degassed aqueous solution. The reason for this, apparently, is that water, in contrast to methylene chloride, during the solvation of luminophoric iridium complexes increases the contribution of nonradiative relaxation of their excited states. A lower PL quantum yield in aqueous solutions compared to solutions in methylene chloride was previously observed for a number of mono- and binuclear cyclometalated iridium (III) complexes [40,41].

It is well known that oxygen is an active quencher of the phosphorescence of cyclometalated iridium complexes [42]. For this reason, the PL quantum yields of **P1**–**P4** polymers in aerated methylene chloride solutions are significantly lower than in degassed solutions (Table 1). The quantum yields in aerated aqueous solutions also differ from those in degassed solutions; however, they differ to a lesser extent than the quantum yields in methylene chloride solutions. The reason for this is the greater solubility of oxygen in methylene chloride than in water [42]. In addition, it can be assumed that, upon the formation of micelles by polymers **P1**–**P4** in an aqueous medium, the hydrophilic shell noticeably limits the penetration of oxygen molecules to the luminophoric iridium complexes located in the micelle core, and as a result, the quenching of the PL of the polymers in aqueous solutions occurs to a much lesser extent.

**Figure 6:**
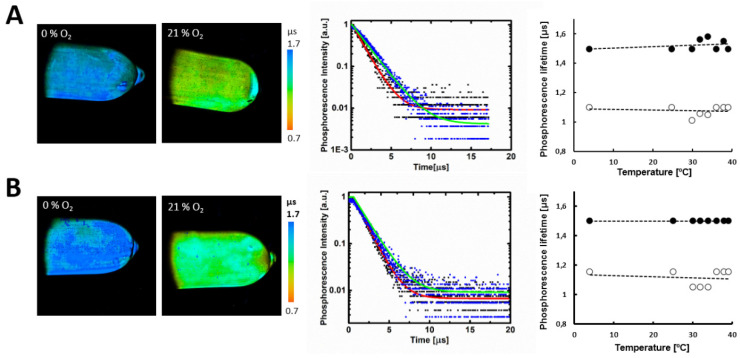
Phosphorescence lifetime measurements for polymers **P1** (**A**) and **P3** (**B**) in degassed and aerated aqueous solutions. λ_ex_ = 375 nm. Left, PLIM images of the tubes with polymer solutions at room temperature. Middle, representative phosphorescence decay curves. Single exponential fits for degassed (green lines) and aerated (red lines) solutions. Right, dependence of phosphorescence lifetime on the temperature in the range 4–39 °C. Black circles: τ_0_, white circles: τ.

For polymers **P1** and **P3**, which exhibit the highest quantum yields, the lifetimes of the excited states in degassed (τ_0_) and aerated (τ, at air equilibrium) aqueous solutions were determined using phosphorescence lifetime imaging (PLIM). Under excitation at the wavelength λ_ex_ = 375 nm, the values of τ_0_ and τ for **P1** are 1.5 μs and 1.1 μs, respectively, and for **P3** 1.5 μs and 1.3 μs (Figure 6, Table 2). Figure S3 displays Stern–Volmer plots for polymers **P1** and **P3** in distilled water for the excitation wavelength 375 nm. Given that the decay curve of phosphorescence is described by a single-exponential decay, the Stern–Volmer equation is valid:

(1) $$\frac{1}{\tau }=\frac{1}{\tau_{0}}+k_{q}pO_{2}$$

where τ_0_ is the phosphorescence lifetime of the polymer at the deoxygenated condition, k_q_ is the bimolecular quenching rate constant and *p*O_2_ is the partial oxygen pressure. From the curves, we found the quenching constants to be 1500 mmHg^−1^s^−1^ and 625 mmHg^−1^s^−1^ for **P1** and **P3**, respectively. The O_2_ sensitivity is higher for **P1** than for **P3** owing to the larger value of the quenching constant. The phosphorescence lifetimes of these polymers are shorter than those (10–100 μs) of Pt(II)-based complexes, but longer than those (<1 μs) of typical Ru(II) complexes used as O_2_ sensors [29]. Thus, these polymers are in the middle in terms of the sensitivity between Pt(II) and Ru(II) complexes. The consequence is that when fast measurements have to be performed, it is better to use polymers **P1** and **P3** than Pt-based complexes (for example, when in addition to the oxygen status, the metabolic status of the cells or tissues also has to be evaluated).

Next, measurements of phosphorescence lifetimes were made in the presence of 5% bovine serum albumin (BSA) and at different temperatures between 4 ℃ and 39 ℃. Adding BSA to solutions of polymers at physiologically relevant concentrations did not affect their phosphorescence lifetimes τ (Table 2). With an increase in temperature, the values of the lifetime of **P1** and **P3** were stable both at zero oxygen and in the presence of oxygen (Figure 6).

### 2.3. Biological Properties of Polymers ***P1***–***P4***

**Figure 7:**
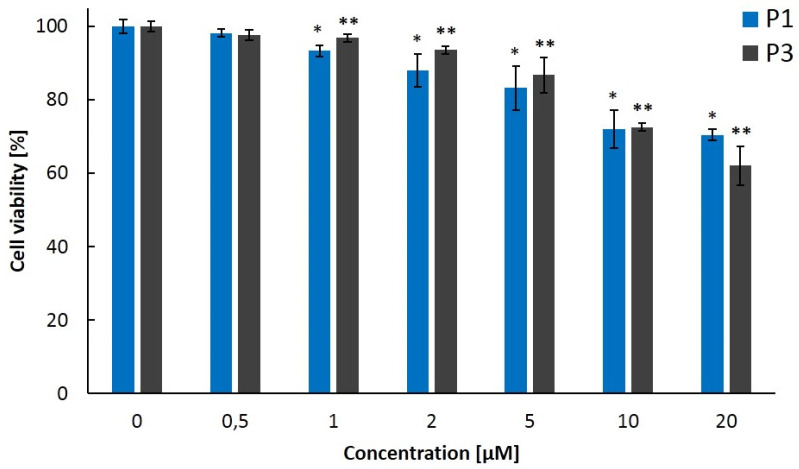
The viability of mouse colon carcinoma cells CT26 after exposure to **P1** or **P3.** The MTT assay was performed in 24 h after adding the polymers to cells. *, ** *p* < 0.05 vs. controls (0 µM). Mean ± SD (n = 3).

To evaluate the cytotoxicity of **P1** and **P3**, the MTT assay was employed (Figure 7). We found that the polymers **P1** and **P3** are not toxic for mouse colon carcinoma cells CT26 at the concentrations 5 µM and below. More than 85% of cells survived after 24 h incubation with these concentrations of the polymers. A decrease in cell viability by 28% (*p* ≤ 0.02) was observed after 24 h incubation with 10 µM of **P1** or **P3**.

**Figure 8:**
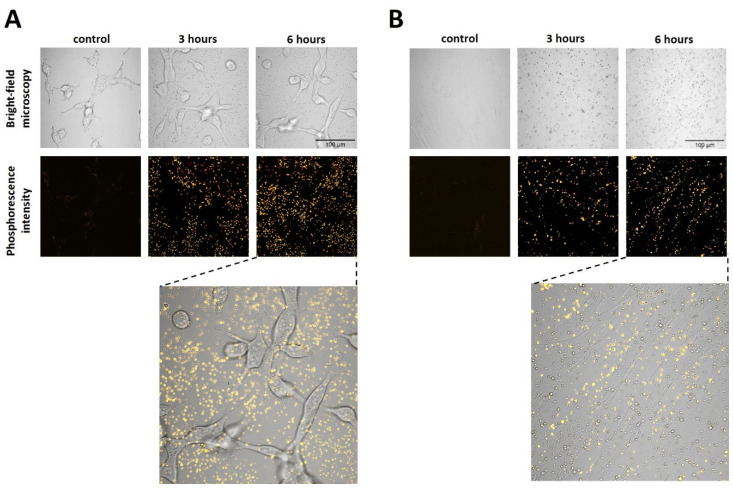
In vitro assessment of cellular uptake of **P3** using laser scanning microscopy. Bright-field microscopy and luminescence intensity images of live mouse colon carcinoma cells CT26 (**A**) and human skin fibroblasts (**B**) exposed to 2.5 µM **P3**. Enlarged images are an overlap of the bright-field with luminescence image for the 6 h time point. Ex. 405 nm, reg. 650–750 nm.

Next, we assessed the ability of the polymers **P1** and **P3** to penetrate into cultured cancer cells and normal fibroblasts (Figure 8). Cellular uptake was investigated by detecting luminescence signals with laser-scanning microscopy. The polymers **P1** and **P3** were added to the cells at non-toxic concentrations and luminescence was monitored for 6 h. We showed that **P3** did not enter into cancer cells or fibroblasts. The signal came only from the culture medium. For **P1**, we failed to register luminescence at the experimental conditions used. Previously, a series of Pd- and Pt-based phosphors with greatly restricted ability to penetrate biological membranes was designed by S. Vinogradov’s group [43,44,45]. Although such probes are not applicable for intracellular oxygen measurements, they can quantify oxygen in living tissues when delivered into vasculature or in the interstitial space [46,47].

Therefore, iridium-containing polymeric micelles **P1** and **P3** in aqueous solutions exhibit sensitivity to oxygen, are cell-membrane-impermeable, and their phosphorescence lifetimes are not altered by albumin and temperature at a physiological range, which makes them potentially suitable for oxygen measurements in biological tissues.

**Figure 9:**
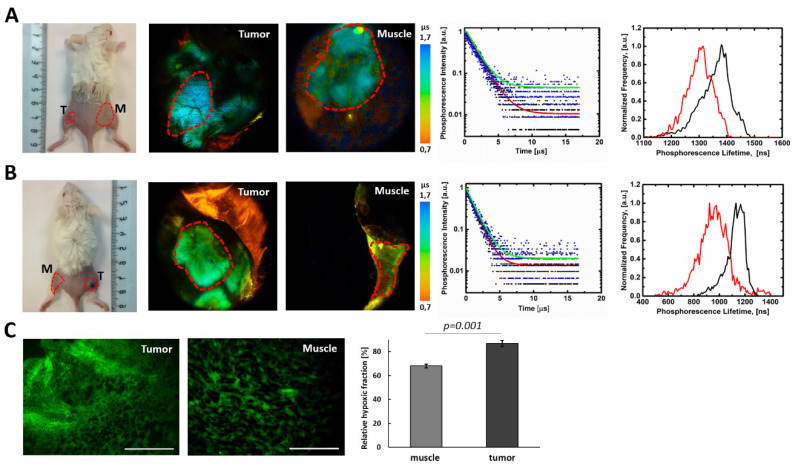
In vivo PLIM of **P1** (**A**) and **P3** (**B**) in subcutaneous mouse colorectal tumor CT26 and muscle. Photographs of mice, representative PLIM images of tumor and muscle after local injection of the polymers. Tumor (T) and muscle (M) areas that were injected with the polymers are shown by the red dashed circles. Phosphorescence decay curves. Single exponential fits for tumor (red lines) and muscle (green lines) in the selected spot of PLIM image. The distribution of the phosphorescence lifetimes for **P1** (upper image, right column) and **P3** (lower image, right column). Red color: muscle. Black color: tumor. **P1**: τ_m_ = 1.3 ± 0.05 µs (muscle); τ_m_ = 1.38 ± 0.06 µs; **P3**: τ_m_ = 0.96 ± 0.11 µs (muscle); τ_m_ = 1.12 ± 0.08 µs (tumor). (**C**) Immunofluorescent staining of tumor and muscle for hypoxia (pimonidazole) and quantification of hypoxic fraction. Scale bar, 400 µm. Mean ± SEM (n = 3–4).

To validate **P1** and **P3** for in vivo applications in animal studies, we measured their phosphorescence lifetimes in tumor and normal tissue (muscle) of a live mouse using PLIM (Figure 9). In a preliminary experiment, the polymers (100 µM, 3 mg/kg in 30 µL PBS) were injected intravenously. However, this dose of the polymer was insufficient to register phosphorescence in the CT26 tumor growing on the mouse thigh. Accordingly, to avoid any toxic effects upon systemic administration of a higher concentration of the polymer, we decided to inject it into the tissues topically. The injection of **P1** and **P3** directly into the mouse tumor or muscle (100 µM, 5 mg/kg in 50 µl PBS) resulted in detectable phosphorescence upon excitation at 375 nm. As expected, phosphorescence lifetimes of both polymers were longer in the tumor than in muscle, as tumors are known to have reduced oxygen levels. The polymer **P1** showed phosphorescence lifetimes of 1.42 ± 0.03 µs in the tumor and 1.06 ± 0.03 µs in the muscle (*p* = 0.02). For **P3**, the lifetime values were 1.33 ± 0.07 µs for the tumor area and 0.87 ± 0.03 µs for the muscle (*p* = 0.02).

To confirm that the increased lifetimes in the mouse tumors are due to decreased oxygen content, the tissues were stained for hypoxia. Immunohistochemical assessment with the established marker pimonidazole revealed a higher level of hypoxia in the tumors than in the muscle, with relative hypoxic fraction 87% vs. 68%, respectively, in the muscle (*p* = 0.001).

The difference between oxygen content in tumors and muscles has been shown using phosphorescence imaging in several papers, including our own [48,49,50,51,52].

Comparison of the lifetimes measured in mouse tissues in vivo with those in solutions shows that the polymers have slightly decreased phosphorescence lifetimes when administrated into tissues, which does not allow the direct conversion of the lifetime values into *p*O_2_ using calibration on solutions. Translating the lifetime values into *p*O_2_ of biological tissue is not a trivial task and requires the calibration conditions to maximally reproduce those ones in the biological system. Even if the calibration is not performed, the distribution of the phosphorescence lifetimes provides information about relative oxygen content and its changes.

Note that all the measurements in vivo were implemented with the excitation λ_ex_ = 375 nm. Since blue light used for one-photon excitation of the polymers has very low penetration depth in tissues, this limits the oxygen mapping by superficial cellular layers, and further work is required to shift the excitation spectra of the polymers to red.

During the last decade, a number of ROMP polymers for biomedical application were synthesized. The preparation and properties of representative examples of such polymers are described in references [37,53,54,55,56,57,58,59,60,61]. The ROMP polymers containing organic dies and anticancer drugs were successfully used as efficient bioimaging and drug-delivery agents [37,55,57,59,62]. The presence of luminescent fragments in macromolecules allowed the monitoring of drug delivery to the target tumor tissue and the evaluation of the efficacy of treatment.

Metabolic processes in cancer cells and tissues are characterized by enhanced oxygen consumption [25,26]. Along with impaired oxygen delivery, this leads to the development of hypoxia that is strongly associated with malignant progression and resistance to therapies. Therefore, oxygen status is of principal importance for the prognosis of oncological diseases.

The oxygenation level in live cells and tissues can be determined by different methods [27,63], and among them optical sensing using phosphorescent transition metal complexes is one of the most suitable because it is noninvasive and allows the evaluation of oxygen content variations rapidly and reversibly [22,27,64]. The applicability of some phosphorescent metal complexes as sensors of oxygen in biological objects is noticeably restricted by their low solubility in water and biological medium. To overcome this disadvantage, two synthetic approaches have been developed. The first one consists of the incorporation of such complexes into water-soluble polymeric micelles [65,66,67]. According to the second approach, the water solubility of phosphorescent metal complexes is provided by the attachment of hydrophilic groups to the ligands bonded to the metal center [43,45,68].

In the paper by Dmitriev et al., 2015, oxygen sensing polymeric probes containing luminescent metal complexes in the main chain were prepared by two-step synthesis consisting of Suzuki coupling copolymerization of organic monomers and monomers containing phosphorescent metal complexes at the first step and the functionalization of the resulting polymer by pendant hydrophilic groups [69]. Zheng et al. synthesized an iridium-containing polymeric probe for cellular and in vivo oxygen sensing by the attachment of a phosphorescent iridium complex to the end group of poly(N-vinylpyrrolidone) [8]. Luminescent water-soluble metal-containing polymers synthesized by the ROMP method were found to be applicable for protein detection [9].

As mentioned above, the determination of oxygen status in cells and tissues is of great importance in biomedical studies aiming to develop new and more efficient methods of diagnosis and treatment of oncology diseases. Among the known approaches of the evaluation of oxygen content in live cells and tissues, the most attractive one consists of the use of noninvasive optical probes. Currently, different phosphorescent transition metal complexes were found to be efficient as oxygen-sensing probes. The variation in oxygen content in cells and tissues can be easily determined by the changing of the emission intensity and phosphorescence lifetimes induced by molecular oxygen quenching. Iridium(III) complexes are the most promising probes for the evaluation of oxygen status in biological objects because of their photostability, readily tunable emission color, high quantum yields and relatively short emission lifetimes. A number of oxygen probes based on luminescent iridium(III) complexes have been developed [22,27,28,29,30,31]. Amphiphilic polymers with chemically bonded phosphorescent iridium(III) complexes are attractive oxygen probes because the hydrophilic units of these polymers prevent the interaction of hydrophobic phosphorescent units with different biomolecules of the biological object and minimize the distortion of oxygen content evaluation. Several examples of such polymers have been synthesized by free-radical polymerization. The ROMP method is widely used for the preparation of different polymeric materials including polymers for biomedical application. Therefore, we attempted to apply ROMP for the synthesis of iridium-containing polymers that can be used as sensors of oxygen in live cells and tissues.

In the present study, we demonstrate for the first time that easily prepared water-soluble ROMP polymers are a promising chemical platform for the generation of phosphorescent oxygen sensors on the basis of iridium(III). Upon further improvements of spectral properties of the synthesized polymers and accurate calibration in biologically relevant conditions, they can be successfully used for the imaging and quantification of oxygen levels in biological tissues in vivo.

## 3. Materials and Methods

### 3.1. Instruments and Characterization

^1^H NMR spectra were recorded using Bruker DPX-200 [^1^H NMR (200 MHz)] and Bruker Avance III-400 [^1^H NMR (400 MHz)] spectrometers. Chemical shifts are reported relative to the signal of residual protons of the deuterated solvent.

IR spectra were registered using a FTIR FSM 1201 spectrometer. The polymer samples were prepared as thin films on KBr pellets.

Molecular weight distributions were determined using gel permeation chromatography (GPC) using a Knauer chromatograph with Smartline RID 2300 differential refractometer as the detector, with a set of two Phenomenex columns (Phenogel sorbent with pore diameter from 104 to 105 Å, eluent–THF, 2 mL/min, 40 °C). The columns were calibrated using 13 polystyrene standards.

The C, H, N and S elemental analyses were performed at the microanalytical laboratory of IOMC on a Elementar Vario EL cube elemental analyzer.

The sizes of the polymer particles in the aqueous solution were determined from the dynamic light scattering data (a Brookhaven NanoBrook Omni device).

Electronic absorption spectra of polymers in CH_2_Cl_2_ and H_2_O were registered using a Perkin Elmer Lambda 25 spectrometer. Photoluminescence spectra were registered using a Perkin Elmer LS 55 spectrometer. Quantum yields of the photoluminescence of **P1**–**P4** in CH_2_Cl_2_ and H_2_O were determined at room temperature, λ_ex_ 360 nm. Quantum yields for polymeric products were calculated relative to Rhodamine B in ethanol (Φf 0.70) [70] as described in [71].

Phosphorescence measurements of the polymers were performed using a confocal scanning macro-PLIM system equipped with a TCSPC-based module (Becker & Hickl GmbH, Berlin, Germany) described in detail elsewhere [72,73]. Briefly, the system consists of a confocal scanner with an object placed in its intermediate image plane. The signal is collected in epi-luminescence mode with a scan lens of 40 mm focal length, sent to the hybrid detector HPM-100-40 and processed with a single photon counting card SPC-150 (Becker & Hickl GmbH, Berlin, Germany). The system allowed us to obtain phosphorescence lifetime maps from areas as large as 18x18 mm with a lateral resolution of around 15 µm.

Phosphorescence was excited in one-photon mode with picosecond diode lasers at 375 nm (BDL-SMN-375, Becker & Hickl GmbH, Berlin, Germany) and 488 nm (BDL-SMN-488, Becker & Hickl GmbH, Berlin, Germany) and detected in the range 584–676 nm. Long pass filters (HQ435LP and ET 495LP, Chroma, Burlington, VT, USA) and a bandpass filter (HQ630/92, Chroma, Burlington, VT, USA) were used in the detection path.

The PLIM data were processed using SPCImage 8.0 software (Becker & Hickl GmbH, Berlin, Germany). The phosphorescence decay curves were fitted with a single-exponential decay model with an average goodness of the fit < 1.2. The average number of photons per curve was >5000. Image collection time was 60 s.

### 3.2. Synthesis of Polymers

General comments. The reagents and solvents were purchased from commercial sources and used without further purification. All reactions with air- or moisture-sensitive substances were performed using the standard Schlenk technique in vacuum or under an argon atmosphere. Organic monomers **1** [74], **2** [36], iridium-containing monomers **3** [34], **4** [35] and 3rd generation Grubbs catalyst (H_2_IMes)(3-Brpy)_2_Cl_2_Ru=CHPh [75,76] were prepared as described in the literature.

*Synthesis of polymer* **P1**. Organic monomer **1** (0.1545 Γ, 0.324 mmol) in 1 mL of THF was placed into an evacuated ampule. Iridium-containing monomer **3** (0.007 g, 0.008 mmol) in 0.5 mL of THF was added to the solution of monomer **1** and finally 0.0027 g (0.003 mmol) of third-generation Grubbs catalyst in 0.5 mL of THF was added to the mixture of monomers **1** and **3** at room temperature. The solution of monomers and catalyst was magnetically stirred at 40 °C. The reaction was monitored by thin-layer chromatography and it was found that polymerization was completed in 10 h. The reaction mixture was cooled to room temperature and after the addition of a few drops of ethyl vinyl ether was stirred for 30 min. The reaction solution was added drop-wise into stirring hexane (40 mL) and precipitated polymeric product was isolated by centrifugation and dried in vacuum at room temperature to a constant weight. The yield of polymer **P1** (red gummy substance) was 0.143 g (88%). Anal. Found: C, 55.70; H, 7.49; N, 0.29. Calcd for C_928_H_1477_N_4_O_442_ Ir: C, 55.86; H, 7.46; N, 0.28. IR, ν, cm^−1^: 2958, 2883 (C_alif_–H); 1746 (C=O); 1452, 1352, 1287, 1202, 1117 (C–O), 983, 858, 758, 519. ^1^H NMR (200 MHz, CDCl_3_, δ, ppm): 9.20–8.80 m (2H), 8.70–8.05 m (4H), 8.00–7.55 m (9H), 7.50–6.80 m (6H), 6.75–6.25 m (4H), 5.93–5.76 m (40H), 5.66–5.43 m (41H), 5.25–4.93 m (41H), 4.78–4.58 m (40H), 4.45–4.10 m (160H), 3.75–3.59 m (642H), 3.58–3.49 m (161H), 3.36 br. s. (240H), 3.10 br. s. (83H), 2.50–1.85 m (2H), 1.40–0.85 m (2H). *M*_w_ = 123,300, *M*_n_ = 63,700, *M*_w_/*M*_n_ = 1.94.

*Synthesis of polymer***P2**. The synthesis of polymer **P2** was carried out as described for polymer **P1**. Yield 0.114 g (86%). Anal. Found: C, 55.25; H, 7.58; N, 0.30; S, 0.26. Calcd for C_924_H_1473_N_4_O_442_ IrS_2_: C, 55.58; H, 7.44; N, 0.28; S, 0.32. IR, ν, cm^−1^: 2953, 2883, 2823 (C_alif_–H); 1741 (C=O); 1452, 1352, 1287, 1202, 1117 (C–O), 1033, 983, 853, 758, 524. ^1^H NMR (200 MHz, CDCl_3_, δ, ppm): 9.10–8.60 m (2H), 8.55–8.05 m (3H), 8.00–7.55 m (9H), 7.50–7.05 m (3H), 7.00–6.55 m (2H), 6.50–6.20 m (2H), 5.96–5.80 m (40H), 5.64–5.44 m (41H), 5.18–4.96 m (41H), 4.78–4.54 m (40H), 4.30–4.10 m (160H), 3.75–3.59 m (642H), 3.56–3.50 m (161H), 3.36 br. s. (241H), 3.10 br. s. (82H), 2.60–2.10 m (2H), 1.00–0.60 m (2H). *M*_w_ = 99,800, *M*_n_ = 48,700, *M*_w_/*M*_n_ = 2.05.

*Synthesis of polymer* **P3**. Organic monomers **1** (0.1510 g, 0.31 mmol) and **2** (0.0447 g, 0.1 mmol) in 1 mL of THF were placed into an evacuated ampule. Iridium-containing monomer **3** (0.0095 g, 0.011 mmol) in 0.5 mL of THF was added to the solution of organic monomers and finally 0.003 g (0.0034 mmol) of third-generation Grubbs catalyst in 0.5 mL of THF was added to the mixture of monomers **1**, **2** and **3** at room temperature. The solution of monomers and catalyst was magnetically stirred at 40 °C. The reaction was monitored by thin-layer chromatography and it was found that polymerization was completed in 10 h. The reaction mixture was cooled to room temperature and after the addition of a few drops of ethyl vinyl ether was stirred for 30 min. The reaction solution was added drop-wise into stirring hexane (40 mL) and precipitated polymeric product was isolated by centrifugation and dried in vacuum at room temperature to a constant weight. The yield of polymer **P3** (red gummy substance) was 0.18 g (88%). Anal. Found: C, 56.26; H, 7.58; N, 1.52. Calcd for C_928_H_1457_N_24_O_402_Ir: C, 56.94; H, 7.50; N, 1.72. IR, ν, cm^−1^: 3327 (N–H); 2953, 2878 (C_alif_–H); 1746, 1681 (C=O); 1536 (N–H); 1452, 1357, 1287, 1252, 1202, 1112, 1033 (C–O); 978, 853, 753, 544. ^1^H NMR (200 MHz, CDCl_3_, δ, ppm): 9.20–8.80 m (2H), 8.70–8.05 m (4H), 8.00–7.55 m (9H), 7.50–6.80 m (6H), 6.75–6.25 m (4H), 6.04–5.78 m (40H), 5.74–5.34 m (41H), 5.32–4.92 m (41H), 4.86–4.42 m (40H), 4.42–4.06 m (140H), 3.81–3.59 m (540H), 3.58–3.47 m (121H), 3.46–3.27 m (181H), 3.22–2.96 m (82H), 2.52–2.02 m (2H), 1.98–1.40 m (62H), 1.05–0.75 m (122H). *M*_w_ = 97,100, *M*_n_ = 54,900, *M*_w_/*M*_n_ = 1.77.

*Synthesis of polymer* **P4**. The synthesis of polymer **P4** was carried out as described for polymer **P3**. Yield 0.128 g (87%). Anal. Found: C, 56.60; H, 7.60; N, 1.80; S, 0.32. Calcd for C_924_H_1453_N_24_O_402_IrS_2_: C, 56.66; H, 7.48; N, 1.72; S, 0.33. IR, ν, cm^−1^: 3322 (N–H); 2953, 2878, 2818 (C_alif_–H); 1741, 1681 (C=O); 1536 (N–H); 1452, 1352, 1287, 1252, 1202, 1112, 1033 (C–O); 978, 848, 753, 534. ^1^H NMR (200 MHz, CDCl_3_, δ, ppm): 9.10–8.60 m (2H), 8.55–8.05 m (3H), 8.00–7.55 m (9H), 7.50–7.05 m (3H), 7.00–6.55 m (2H), 6.50–6.20 m (2H), 5.96–5.80 m (40H), 5.64–5.44 m (41H), 5.18–4.96 m (41H), 4.78–4.54 m (40H), 4.30–4.10 m (140H), 3.75–3.59 m (542H), 3.59–3.49 m (121H), 3.36 br. s. (181H), 3.10 br. s. (82H), 2.55–2.00 m (2H), 1.90–1.40 m (60H), 1.05–0.75 m (122H). *M*_w_ = 80,600, *M*_n_ = 51,600, *M*_w_/*M*_n_ = 1.56.

### 3.3. In Vitro Cytotoxicity Assay and Cellular Uptake Study

Cell viability was determined by the MTT assay that measures the reduction of substrate 3-(4,5-dimethylthiazol-2-yl)-diphenyltetrazolium bromide (MTT) to a dark-blue formazan product by mitochondrial dehydrogenases in living cells. A total of 2 × 10^3^ CT26 cells in 100 mL of culture medium DMEM were seeded onto 96-well plates and incubated at 37 °C, 5% CO_2_. The cells were allowed to attach overnight; then, **P1** or **P3** was added at the concentrations from 0.5 µM to 20 µM for 24 h. The MTT reagent (Alfa Aesar, Haverhill, MA, USA) was added to the culture medium in the final concentration of 0.5 mg/mL for 4 h. The absorbance was measured at 570 nm with a multimode microplate reader (Synergy Mx; BioTek Instruments, Winooski, VT, USA). The proportion of viable cells was calculated as the percentage of purple formazan-stained treated cells to that of the untreated control cells. All assays were performed in triplicate.

Laser scanning microscopy was used to analyze the cellular uptake of **P1** and **P3** in cultured cells. Luminescence images were obtained with an LSM 880 laser scanning microscope (Carl Zeiss, Oberkochen, Germany). Luminescence was excited at the wavelength 405 nm with a diode laser and detected in the range of 650–750 nm. The average power applied to the samples was ~8 mW. The images were acquired through a water immersion objective C-Apochromat 40×/1.2 NA.

For live-cell confocal microscopy, CT26 cells (2 × 10^5^ cells in 2 mL DMEM) or human skin fibroblasts (2 × 10^5^ cells in 2 mL DMEM) were seeded in glass-bottomed 35 mm FluoroDishes and incubated overnight (37 °C, 5% CO_2_). Then, the cells were washed with phosphate-buffered saline (PBS) and placed in FluoroBright DMEM media (Thermo Fisher Scientific, Waltham, MA, USA) containing 2.5 µM **P1** or **P3**. The uptake was monitored for 6 h.

### 3.4. In Vivo Animal Study

All animal protocols were approved by the Local Ethical Committee of the Privolzhsky Research Medical University (Nizhny Novgorod, Russia). PLIM was performed on Balb/c mice, female, 20–22 g body weight, with subcutaneously inoculated murine colon carcinoma CT26. To generate tumors, mice of 20–22 g body weight were injected subcutaneously in the flank with 5 × 10^5^ CT26 cells in 200 mL PBS. Phosphorescence lifetime measurements were conducted on days 10–12 of tumor growth, when the tumors reached the size of ~7−12 mm in diameter. Mice were anesthetized by intramuscular injection of Zoletil (40 mg/kg, Virbac SA, Carros, France) and 2% Rometar (10 mg/kg, Spofa, Prague, Czech Republic).

**P1** and **P3** were dissolved in distilled water in a concentration 2 mg/ml and injected into the tumor 5–15 min prior to the PLIM study (50 µL of solution in 4–6 injections, total dose 5 mg/kg body weight). Similar injection was performed in the muscle on the opposite leg to measure phosphorescence lifetime in the muscle.

### 3.5. Immunohistochemical (IHC) Staining for Hypoxia

The hypoxia marker pimonidazole hydrochloride (Hypoxyprobe-1, Chemicon International, Temecula, CA, USA) was injected in mice intravenously at the dose of 60 mg/kg. After 40 min, the mice were sacrificed by cervical dislocation, and the tumors were frozen in OCT mounting medium. Seven-micrometer-thick cryosections were subsequently stained with FITC-conjugated IgG1 mouse monoclonal antibodies diluted 1:100, according to the manufacturer’s protocol. The entire tumor cross sections were then imaged on a fluorescent microscope Leica DMIL LED (Wetzlar, Germany), ex. 488 nm, reg. 500–540 nm. Relative hypoxic fraction was calculated in ImageJ software (NIH, Bethesda, MD, USA) as the percentage of pimonidazole-positive area divided by the total tumor area.

## 4. Conclusions

In summary, new water-soluble functionalized polynorbornenes containing oligoether groups, amino acid fragments and luminophoric complexes of iridium(III) in the side chains were designed. These polymers are easily prepared by the ROMP method and exhibit intense oxygen-dependent red photoluminescence, good solubility in water and membrane impermeability, which make them promising agents for oxygen sensing in tissues. We present preliminary in vivo data from an animal model that provide evidence of the potential applicability of the new iridium-containing polymeric micelles as phosphorescent oxygen probes. In general, the physicochemical, photophysical and biological properties of the synthesized polymers allow us to consider them as promising optical sensors of oxygen in organic solvents, water and in biological objects.

## Figures and Tables

**Table 1:** Photophysical characteristics of polymers **P1**–**P4**.

Polymer	λ_max_^abs^/nm (log ε) in CH_2_Cl_2_	λ_max_^em^/nm (CH_2_Cl_2_)	Quantum Yield, % (in CH_2_Cl_2_)		Quantum Yield, % (in Water)		Chromaticity Coordinates in the CIE Diagram (*x*; *y*)
			(a)	(b)	(a)	(b)	
**P1**	297 sh (4.29), 344 (3.97), 476 (3.60), 550 (3.26)	618	10.0	2.7	3.8	2.8	0.66; 0.34
**P2**	300 sh (4.45), 353 (4.29), 472 (3.91), 525 (3.90)	657	2.5	0.5	0.5	0.3	0.71; 0.29
**P3**	299 sh (4.08), 346 (3.75), 477 (2.95), 552 (2.59)	618	9.3	3.6	2.7	2.0	0.66; 0.34
**P4**	302 sh (4.18), 353 (4.04), 471 (3.66), 527 (3.65)	657	2.6	0.5	0.4	0.2	0.71; 0.29

^a^ Degassed solution. ^b^ Aerated solution.

**Table 2:** Phosphorescence lifetimes of polymers **P1** and **P3** in degassed (τ_0_) and aerated (τ) aqueous solutions at room temperature. The values of τ in 5% BSA are shown in brackets, λ_ex_ = 375 nm.

	P1	P3
τ_0,_ μs	1.5 ± 0.07	1.5 ± 0.08
τ, μs (τ in BSA, μs)	1.1 ± 0.06 (1.2 ± 0.07)	1.3 ± 0.06 (1.2 ± 0.06)
Dynamic range r = τ_0_/τ	1.36 ± 0.072	1.15 ± 0.07
Photon count rate (for τ)	5890	5600

## Data Availability

Not applicable.

## Supplementary Materials

The following are available online. Figure S1: ^1^H NMR spectra of polymers **P2**, **P4** in CDCl_3_. (*) Signal derived from the solvent; Figure S2: Absorption spectra of **P1**, **P2** (a) and **P3**, **P4** (b) polymers in H_2_O solution; Figure S3: Stern–Volmer plot for polymers **P1** and **P3** in H_2_O solution at 375 nm excitation; Table S1: Photophysical characteristics of polymers **P1**–**P4** in H_2_O solution.

## Sample Availability

Samples of the compounds are not available from the authors.
