# Supplementary material for: Red Light-Emitting Water-Soluble Luminescent Iridium-Containing Polynorbornenes: Synthesis, Characterization and Oxygen Sensing Properties in Biological Tissues In Vivo
Source: Molecules. 2021 Oct 20;26(21):6349. doi: 10.3390/molecules26216349 (PMC8587708; doi:10.3390/molecules26216349)
Supplement: Supplementary file 1 [file molecules-26-06349-s001.zip › molecules-1399712-supplementary.pdf]

Supplementary Material for the article

Red Light-Emitting Water-Soluble Luminescent Iridium-Containing Polynorbornenes. Synthesis, Characterization and Oxygen Sensing Properties in Biological Tissues *in Vivo*

Leonid N. Bochkarev [1,*], Yulia P. Parshina [1], Yana V. Gracheva [1], Tatyana A. Kovylina [1], Svetlana A. Lermontova [1], Larisa G. Klapshina [1], Aleksey N. Konev [1], Mikhail A. Lopatin [1], Maria M. Lukina [2], Anastasia D. Komarova [2], Vladislav I. Shcheslavskiy [2,3], Marina V. Shirmanova [2]

[1] G. A. Razuvaev Institute of Organometallic Chemistry, Russian Academy of Sciences, Tropinina, 49, 603950 Nizhny Novgorod, , Russia; jully@iomc.ras.ru (Y.P.P); yanashlyapugina@yandex.ru (Y.V.G.); gluhova@iomc.ras.ru (T.A.K.); lermontovasa@rambler.ru (S.A.L.); klarisa@iomc.ras.ru (L.G.K.); alex-kon@mail.ru (A.N.K.); lopatin@iomc.ras.ru (M.A.L.)

[2] Institute of Experimental Oncology and Biomedical Technologies, Privolzhsky Research Medical University, Minin and Pozharsky Sq. 10/1, 603005 Nizhny Novgorod, Russia; kuznetsova.m.m@yandex.ru (M.M.L.); leeemonk76g@gmail.com (A.D.K.); vis@becker-hickl.de (V.I.S.); shirmanovam@mail.ru (M.V.S.)

[3] Becker&Hickl GmbH, Nunsdorfer Ring 7-9, 12277 Berlin, Germany

* Correspondence: lnb@iomc.ras.ru

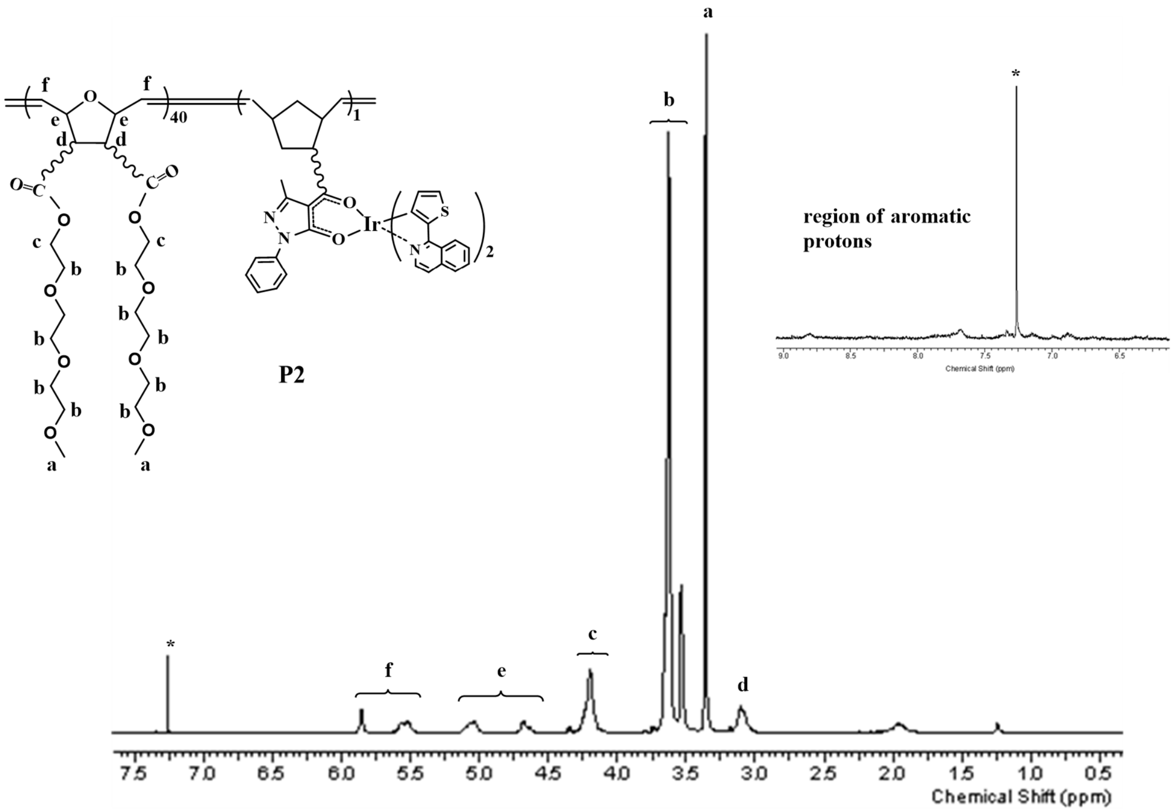

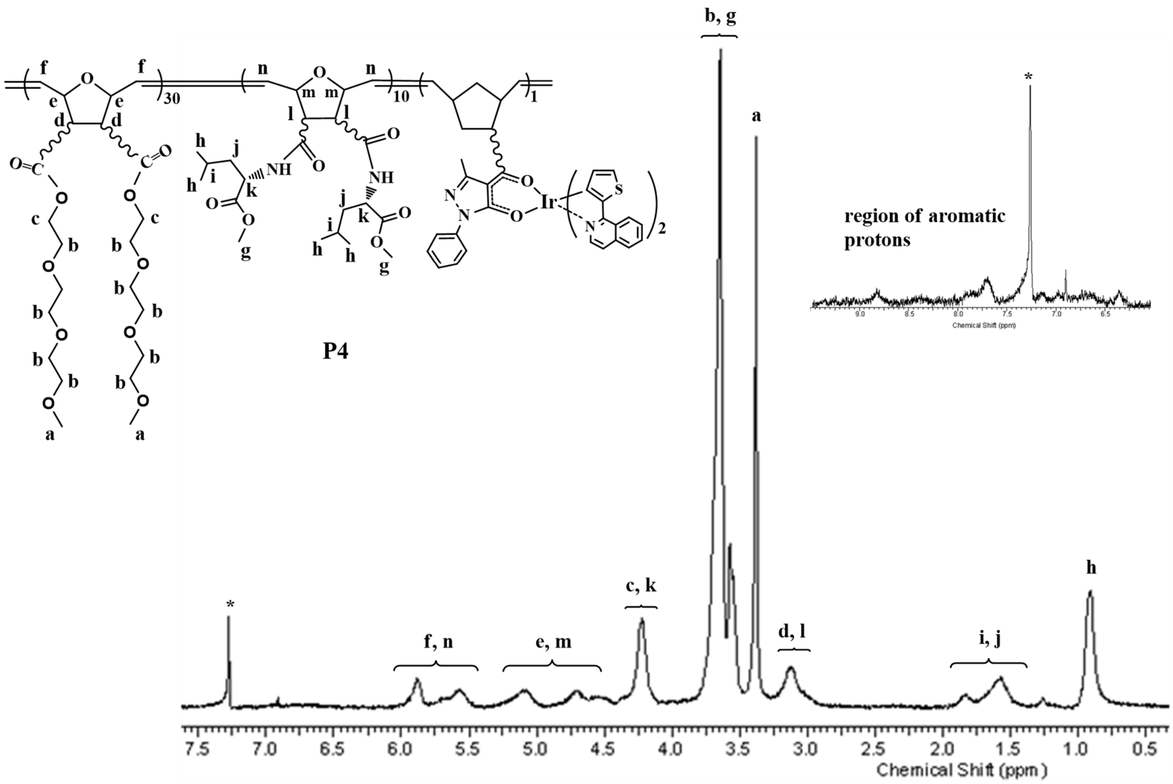

Figure S1. ^{1}H NMR spectra of polymers **P2**, **P4** in $CDCl_3$. (*) Signal derived from the solvent.

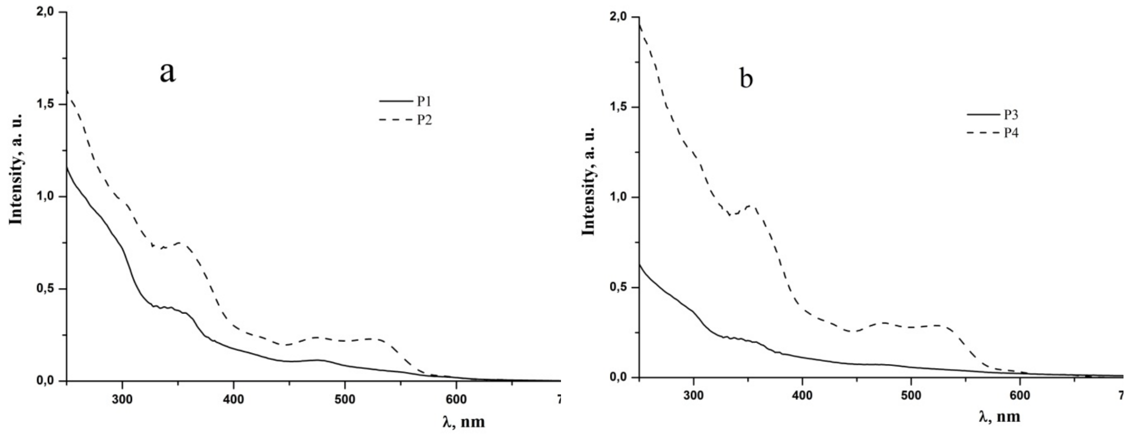

(**a**) (**b**)

Figure S2. Absorption spectra of polymers **P1**, **P2** (a) and **P3**, **P4** (b) in H_2O solution.

Table S1. Photophysical characteristics of polymers **P1**–**P4** in H_2O solution

Polymer	λ_{max}^{abs}/nm (log ε) in H_2O	λ_{max}^{em}/nm (in H_2O)	Quantum yield, % (in H_2O)		Chromaticity coordinates in the CIE diagram (*x*; *y*)
			a)	b)	
**P1**	297 sh (4.60), 344 (4.33), 476 (3.76), 550 (3.42)	618	3.8	2.8	0.66; 0.34

**P2**	300 sh (4.49), 353 (4.38), 472 (3.88), 525 (3.87)	657	0.5	0.3	0.71; 0.29
**P3**	299 sh (4.56), 346 (4.31), 477 (3.83), 552 (3.55)	618	2.7	2.0	0.66; 0.34
**P4**	302 sh (4.49), 353 (4.38), 471 (3.88), 527 (3.86)	657	0.4	0.2	0.71; 0.29

a) Degassed solution.

b) Aerated solution.

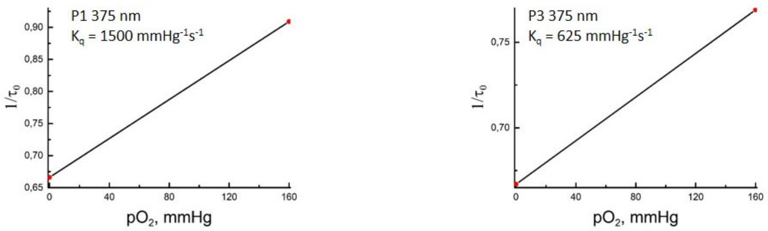

Figure S3. Stern-Volmer plot for polymers **P1** (left image) and **P3** (right image) in H_2O solution at 375 nm excitation.
